# Gallstone ileus treated with non-surgical conservative methods: a case report

**DOI:** 10.1186/1752-1947-9-15

**Published:** 2015-03-02

**Authors:** Alessandro Pezzoli, Antonella Maimone, Nadia Fusetti, Elena Pizzo

**Affiliations:** 5 Department of Gastroenterology and Endoscopy Unit, Sant’Anna University Hospital, v. A. Moro 8 203, 44124 Cona, Ferrara, Italy; 6 Department of Applied Health Research, University College London, 1-19 Torrington Place, London, WC1E7HB UK

**Keywords:** Gallstone ileus, Endoscopy, Extracorporeal shock-wave lithotripsy

## Abstract

**Introduction:**

The preoperative diagnosis of gallstone ileus is challenging due to the variability of its presentation, often resulting in late diagnosis. Controversy remains regarding the management of gallstone ileus; surgery is the standard treatment, but also less invasive approaches have proven to be successful. We present an unusual case of gallstone ileus and its conservative treatment.

**Case presentation:**

We describe the case of a 49-year-old Caucasian woman with a bowel sub-occlusion, treated conservatively. The imaging technique (plain abdominal X-ray and computed tomography scan) led to a diagnosis of gallstones ileus. A surgical intervention was not performed. Instead, she underwent extracorporeal shock-wave lithotripsy to fragment the stones, mechanical intestinal dilatation for ileocolic stenosis and endoscopic removal of the gallstone. The presence of an apricot shell contributed to the bowel occlusion and was removed. The intervention was successful and without complications.

**Conclusions:**

Given the variability of the gallstone ileus presentation, surgery could not be the only treatment for our patient. In our case report, we show that colonoscopy could be a non-invasive approach that allows for diagnosis and treatment at the same time. The available data do not show a higher rate of recurrent biliary disease in cases where this method has been used, therefore in select patients, a conservative treatment could be an effective solution.

## Introduction

Gallstone ileus is a rare mechanical bowel obstruction caused by the transition of a gallstone in the gastrointestinal tract through a biliary-enteric fistula, or following endoscopic retrograde cholangiopancreatography (ERCP), which occurs in 1 to 3% of all cases of mechanical ileus [1]. Our patient's history of gallbladder disease and the development of imaging techniques played a key role in the preoperative diagnosis. The traditional treatment of gallstone ileus is surgery [2], however the surgical mortality rate is high and a less invasive approach, such as endoscopic therapies, could be considered. The success of these techniques greatly depends on the size of the gallstone and the location of the intestinal obstruction. We describe a case of gallstone ileus and how a combination of endoscopic and extracorporeal shock-wave lithotripsy (ESWL) treatment can be an effective solution.

## Case presentation

A 49-year-old Caucasian women presented to our department with a one-day history of nausea, vomiting and abdominal pain. Approximately two months previously, she had presented with symptoms of biliary colic, which was subsequently regressed with non-steroidal anti-inflammatory drugs. Her medical history included caecum and terminal ileum resection for bowel ischemia with ileocolic anastomosis. On examination she did not present with any signs of intestinal obstruction or peritonitis and her rectal examination was normal. The laboratory findings, including her white blood count, C-reactive protein (CRP), aspartate aminotransferase (AST), alanine aminotransferase (ALT), Υ-glutamyl transpeptidase (GGT), alkaline phosphatase (AP) and bilirubin levels, were within the normal range.

**Figure 1:**
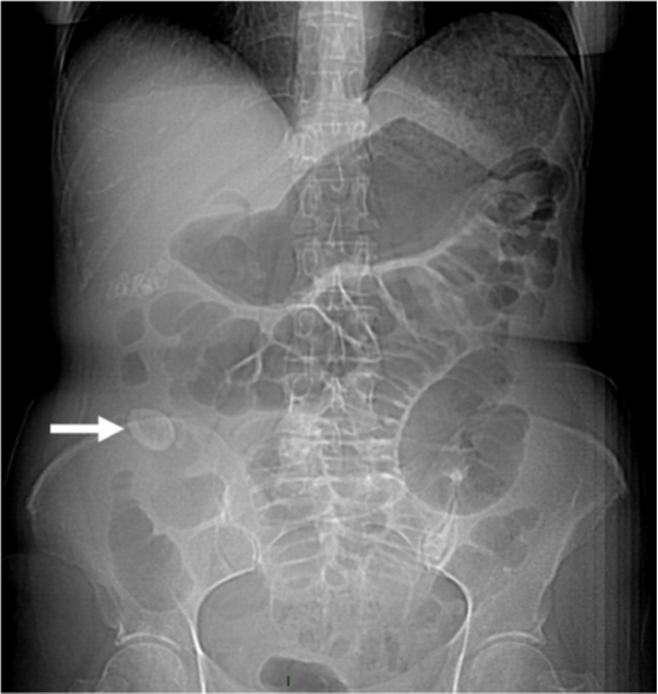
**Plain abdominal X-ray showing an obstruction and dilated intestinal loops.** Arrow indicates gallstone or possibly the apricot shell.

Her abdominal radiographs showed dilated loops and air-fluid levels, mainly in the terminal ileum, however the rectal ampulla was inhabited by stool and air. Calcified masses were identified in the right upper quadrant (Figure 1).

**Figure 2:**
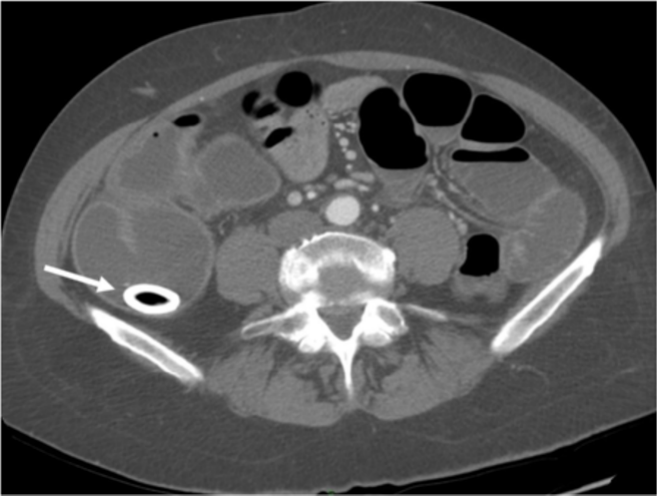
**Computed tomography scan showing the stone in the terminal ileum.** Arrow indicates gallstone.

**Figure 3:**
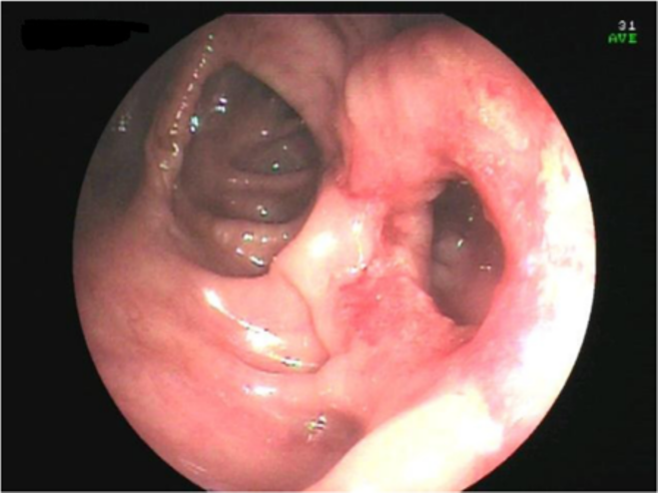
**Ileocolonic anastomosis after pneumatic dilatation.**

**Figure 4:**
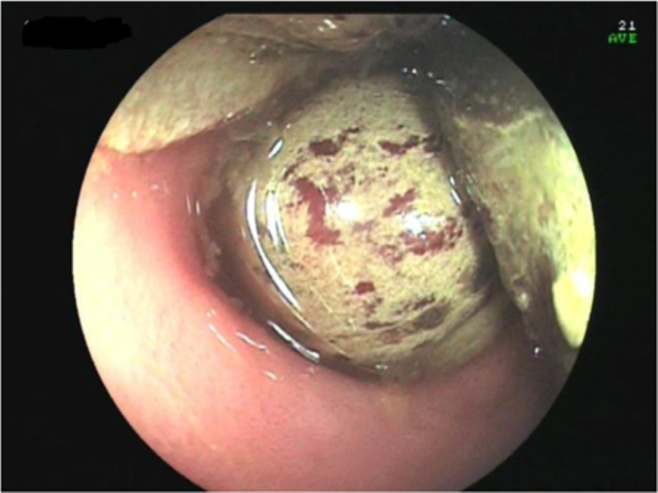
**Stones in the small bowel above the anastomosis.**

**Figure 5:**
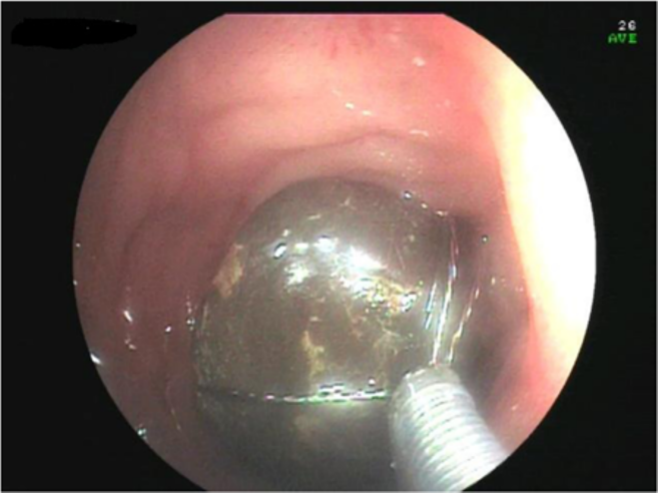
**Stone retrieved with a Dormia basket.**

**Figure 6:**
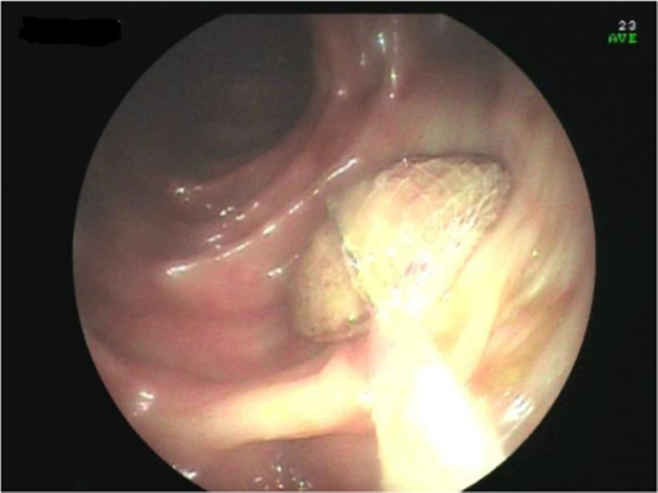
**Apricot shell retrieved with a Roth net.**

A computed tomography (CT) scan of the abdomen confirmed dilatation of her distal small bowel, which was more marked in the pre-anastomotic loop, with a calcified obstructive intraluminal formation (Figure 2). Her gallbladder presented with a high number of stones which were adjoined to her intestinal wall. Therefore the patient underwent a colonoscopy which confirmed an anastomotic sub-stenosis that could not be explored with a standard endoscope. We performed a mechanical dilation with a control radial expansion (CRE) balloon catheter (Boston Scientific Corp Marlborough, USA) up to 18mm in diameter (Figure 3). There were three calcified masses in her small bowel (Figure 4), two of which were recovered using a Dormia basket (Cook Endoscopy, Bloomington, USA) (Figure 5). The third, lager than 20mm, did not exceed the ileocolic anastomosis and therefore was treated with ESWL. She underwent two sessions of ESWL. A 30mm calcified biliary stone was identified just proximal to her ileocolic anastomosis; it was fragmented and removed during a subsequent colonoscopy. Unexpectedly, after the stone removal, an apricot shell was identified and retrieved with a Roth net retriever (US Endoscopy, Mentor, USA) (Figure 6). A subsequent magnetic resonance cholangiopancreatography (MRCP) did not find a biliary-enteric fistula or pneumobilia, although there was the presence of gallstones in the small bowel.

She reported a resolution of the occlusive symptoms; the surgeon chose to avoid a cholecystectomy because she was considered a high-risk patient. At six months after discharge she has remained without reoccurrence of symptoms.

## Discussion

Gallstone ileus is a rare bowel obstruction caused by gallstones; the most frequent mechanism is the migration of stones through a gallbladder-duodenal fistula [1–3]. Often gallstone ileus is the consequence of an episode of acute cholecystitis and gallbladder adhesion to the bowel. This condition is more frequent in women, given the known prevalence of gallbladder disease in this gender, and in patients over 65 years. The size of the stone represents a key factor in the development of the disease, as a stone bigger than 2.5cm can cause a bowel obstruction; nonetheless even smaller stones can cause gallstone ileus in cases of stenosis. The gallstone usually impacts the terminal ileus or the ileocecal valve, although colonic gallstones can happen when there is a gallbladder-colon fistula [4]. However, rare cases of gallstone ileus have been reported even in absence of a bilioenteric fistula, as in this study [5]. This can be explained by the entrance of the stone through the Vater papilla. In our case report a bilioenteric fistula was not detected, but the presence of a narrow anastomosis might have helped the growth of the stones. Indeed, the alteration of the bile salts in the enterohepatic circuit following an ileum resection or in patients with Crohn’s disease is a well-known phenomenon [6], and our patient had previously undergone an ileal resection that might have caused the development of the stone.

A preoperative diagnosis of gallstone ileus is challenging. The clinical presentation can be characterized by non-specific symptoms and it usually depends on the localisation and the nature of the obstruction (partial or full). However, in cases of a clinical history of gallstones, clinical signs of cholecystitis and bowel obstruction, gallstone ileus can be strongly suspected. The onset can manifest as acute, intermittent or chronic episodes.

Plain abdominal X-rays, abdominal ultrasound and CT scans may reveal signs of gallstone ileus and help in the preoperative diagnosis and management of this disease. The Rigler’s triad of radiological features consists of mechanical bowel obstruction, pneumobilia and ectopic stone within the intestinal lumen [7]. The presence of the Rigler triad in a plain abdominal radiography varies between 17 and 87%; if present, two out of three signs are considered to be sufficient to establish a diagnosis. Only 15% of gallstones are sufficiently calcified to be visualized as radiopaque in a plain abdominal X-ray or CT. In our case report, this sign was evident but we can speculate that only the bigger stone was radiopaque and that the apricot shell had helped the shock-wave treatment, working as a marker. All other imaging techniques performed, including the MRCP, were not able to clearly identify the fistula or the pneumobilia. A possible explanation for this phenomenon may be found in the modified anatomy of the bowel of our patient that led to an increase in size of the stone, due to the sedimentation of intestinal content; moreover, the presence of bowel stenosis caused the gallstone ileus.

Controversy remains about the management of gallstone ileus. Although spontaneous resolution of gallstone ileus are described [8], it generally causes acute bowel obstruction, and the aim of treatment is gallstone extraction. An enterolithotomy with stone extraction, followed (or not) by elective biliary surgery, is the therapy of choice. No other approach is clearly identified as superior. The one-stage procedure (enterotomy, fistula repair and cholecystectomy) is strongly associated with a greater mortality rate, largely due to a delayed diagnosis and concomitant diseases [9]. On the other hand, performing the biliary surgery (colocistectomy) and the fistula repair at the same time reduces complications related to gallstones disease, including recurrent ileus. The two-stage procedure (enterolithotomy followed by cholecystectomy and repair of the biliodigestive fistula after four to six weeks) is an alternative treatment that is suggested for younger patients and in cases of recurrent biliary symptoms [10]. Laparoscopic procedures can be an alternative method, although a surgeon with specialist experience in advanced laparoscopic surgery would be necessary [11]. The endoscopic treatment of gallstone ileus is a valid alternative approach and some cases have already been reported [12–14]. Ultrasound-guided ESWL has also been suggested as a non-invasive alternative to surgery to fragment the stone and solve the sub-occlusion [3].

In our case report, the nuanced presentation and the possibility to access the obstruction endoscopically suggested a conservative treatment approach. This case shows some different features when compared with cases described elsewhere. First of all, the age of our patient was uncommon, since gallstone ileus is more frequent in patients over 65 years of age [1]. The presence of an apricot shell represents a clinical curiosity, but was also a sign of a difficult enteric transit from the ileocolic anastomosis that could be responsible for the growth of the stones. In fact, a bilioenteric fistula was not diagnosed in our patient. Moreover, she denied having eaten apricots in the most recent months prior to her symptoms and the stone most likely had been there for a considerable time and contributed to the bowel obstruction.

An endoscopic dilatation allowed us to remove two stones but a bigger third stone required an ESWL. We did not have to place a radiopaque marker to guide the ESWL, unlike in a previous case [14], because this time the stone was calcified and clearly visible. Moreover, the apricot shell was well visualized at the X-ray during the ESWL and underwent an ESWL, since it had been misunderstood with a stone. The conservative approach taken in our case of dilatation of ileocolic stenosis resulting in the endoscopic removal of the gallstones successfully resolved the disease. The success of this treatment is linked to the localisation and size of the obstruction. The unusual presence of an apricot shell could have contributed to the intestinal occlusion but, paradoxically, facilitated the treatment of ESWL. Since there is evidence showing that only 10% of patients require secondary biliary surgery [15], in selected patients, a combination of endoscopic and ESWL treatment could give positive results.

## Conclusions

Our case report demonstrates that gallstones may enter the gastrointestinal tract through the Vater papilla and later increase in size. The presence of a narrow tract of intestine can facilitate the incidence of gallstone ileus. Given the variability of gallstone ileus presentation, the treatment may not necessarily require a surgical treatment. In our case report, colonoscopy and ESWL were the non-invasive approaches that allowed for diagnosis and treatment simultaneously. Available data does not show a higher rate of recurrent biliary disease, therefore in selected patients conservative treatment may be a curative therapy.

## Consent

Written informed consent was obtained from the patient for publication of this case report and any accompanying images. A copy of the written consent is available for review by the Editor-in-Chief of this journal.

## Abbreviations

ERCP: Endoscopic retrograde cholangiopancreatography
ESWL: Ultrasound-guided extracorporeal shock wave lithotripsy
CT: Computed tomography
MRCP: Magnetic resonance cholangiopancreatography.
